# Brain Transcriptome Analysis Reveals Exercise Improves Methamphetamine‐Induced Impairments in Mouse Learning and Memory Abilities

**DOI:** 10.1111/adb.70077

**Published:** 2025-08-09

**Authors:** Qiuyue Huang, Jisheng Xu, Xuejie Zhang, Changling Wei, Tianzhen Zheng, Xin Liang, Xue Li

**Affiliations:** ^1^ School of Sports Medicine and Health Chengdu Sport University Chengdu China

**Keywords:** learning and memory abilities, methamphetamine, RNA‐seq

## Abstract

Methamphetamine (METH) abuse can inflict profound and enduring neurotoxic effects on the brain, culminating in cognitive dysfunction and impairment of learning and memory. Physical exercise can stimulate both structural and functional adaptations in the central nervous system. The primary objective of this study was to elucidate the safeguarding effect and underlying mechanisms of treadmill exercise intervention in the brains of METH‐addicted mice. Two‐month‐old adult mice were randomly assigned into three distinct groups: the control group (Group C), receiving intraperitoneal injections of saline; the METH treatment group (Group Ma), exposed to intraperitoneal METH administration; and the exercise group (Group Ea), which underwent a two‐week regimen of treadmill exercise intervention following intraperitoneal METH exposure. The conditioned place preference experiment was executed to evaluate METH addiction. The results showed that both Groups Ma and Ea mice became addicted to METH after METH administration (*p* < 0.05, *n* = 6). In the Y‐maze experiment, the exploration time of mice in Group Ea in the novel arm was significantly higher than that in Group Ma (*p* < 0.05, *n* = 6), indicating that exercise intervention improved the learning and memory capabilities of mice. Subsequently, the mouse brain specimens were harvested for transcriptome sequencing and real‐time fluorescence quantitative PCR analysis (*n* = 3). Transcriptome sequencing analysis identified 316 differentially expressed genes (DEGs) in Group Ma compared to Group C, while 156 DEGs were detected in Group Ea compared to Group Ma. Kyoto Encyclopedia of Genes and Genomes analysis outcomes underscored the substantial association of DEGs, discerned in exercise‐intervention mice compared to METH‐treated mice, with key signalling pathways, notably the PI3K‐Akt, mTOR and Wnt signalling pathways, among others. Cross‐analysis revealed 43 DEGs in exercise‐treated mice, such as NFKBIA, CXCL12 and Vav3. Our results revealed changes in the expression profile of the brain transcriptome of METH‐addicted mice and indicated that treadmill exercise intervention affects the expression changes of the brain transcriptome of METH‐addicted mice. The above research results provide unique insights into the further study of the mechanism of treadmill exercise intervention in improving the learning and memory abilities of METH‐induced mice.

## Graphical abstract

Treadmill exercise improved METH‐induced learning and memory impairment in mice.Transcriptome analysis revealed that exercise modulated key pathways including PI3K‐Akt, mTOR, and Wnt signaling. NFKBIA, CXCL12, and Vav3 were identified as potential mediators of the beneficial effects.

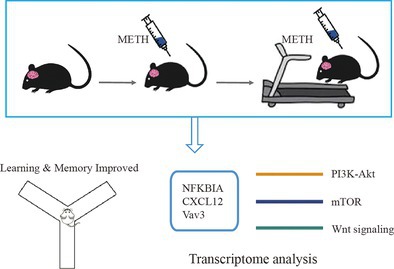

**Abbreviations**
BP: biological processes
CC: cellular components
CNS: central nervous system
CPP: the conditioned place preference
CXCL12: C‐X‐C motif chemokine ligand 12
CXCL4: C‐X‐C motif chemokine receptor 4
DEGs: differentially expressed genes
FPKM: the reads per kilobase of exon per million fragments mapped
GO: Gene Ontology
KEGG: Kyoto Encyclopedia of Genes and Genomes
METH: methamphetamine
MF: molecular functions
NF‐κB: the nuclear factor kappa B
PPI: protein to protein interaction network
RNA‐seq: RNA sequencing
RT‐qPCR: reverse transcription real‐time quantitative PCR

## 1 Background

Methamphetamine (METH) is a highly addictive and prohibited neurostimulant. It is known to cause a range of severe physical, behavioural and psychological issues, including paranoia, hallucinations, violent tendencies, attention deficits, psychosis, anxiety and depression [1]. The global prevalence of METH abuse has engendered profound medical and public health problems. The repercussions of METH addiction in the brain are characterized by detrimental effects, such as oxidative stress, mitochondrial toxicity, excitotoxicity and neuroinflammation, which precipitate perturbations in brain structure and function [2]. The neurological ramifications of METH addiction include impairments in memory, learning and cognitive aptitude [3, 4, 5]. Repeated METH administration is known to cause structural and metabolic alterations across diverse brain regions, including the basal ganglia, hippocampus, amygdala, thalamus and various cortical areas [4]. Notably, changes in the hippocampus and frontal cortex are intimately linked to cognitive deficits and diminished learning and memory capabilities induced by METH.

Physical exercise has been proposed as a potential therapy for METH addiction. Physical exercise can mitigate the symptoms of depression and anxiety, enhance cognitive performance and ameliorate cravings and dependency, attributes commonly observed among both current and abstinent METH users. The neurobiological changes in METH users through physical exercise involve several factors, including the modulation of neurochemicals in the central nervous system (CNS) [6]. Several hypotheses have been proposed to elucidate the neurobiological mechanisms underlying the beneficial effects of exercise in the context of METH addiction. These mechanisms include rectification of neurochemical imbalances, reduction of oxidative stress, stabilization of the blood–brain barrier and restoration of alterations in neurogenesis and gliogenesis among METH users [7]. Nevertheless, the precise mechanisms governing the effect of exercise on METH addiction remain unclear.

As an efficient bioinformatics technology, transcriptome sequencing technology (RNA‐seq) based on a high‐throughput sequencing platform can comprehensively obtain the number and type of transcription products of species‐specific tissues or organs, which is conducive to revealing molecular mechanisms. RNA‐seq has gained prominence across diverse domains, including genomics, genetics and developmental biology. Some previous studies have investigated the transcriptome and epigenetic changes of addicted animals induced by exercise, such as using RNA‐seq to explore the transcriptional profile of wheel running in cocaine addiction [8] and using single nucleus RNA sequencing to clarify the molecular changes caused by wheel running in the hippocampus [9]. In this study, RNA‐seq was used to scrutinize the transcriptome of brain genes in mice with exercise‐induced METH addiction with the aim of offering insights and substantiating the potential of exercise as a therapeutic avenue for addressing METH addiction and its associated learning and memory impairments.

## 2 Results

### 2.1 METH Treatment Leads to Addictive Behaviour in Mice

**FIGURE 1:**
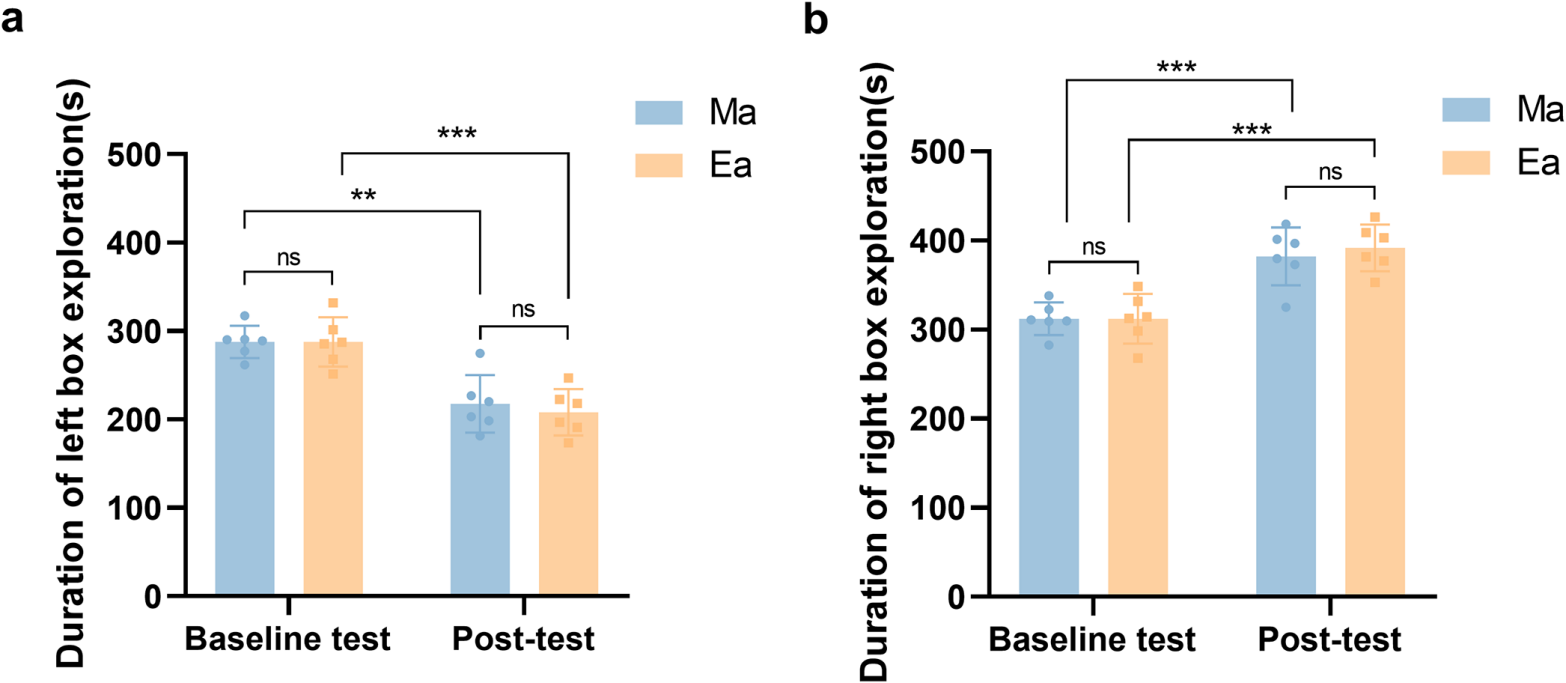
CPP tests were conducted on Groups Ma and Ea after METH injection to assess METH addiction in mice. The left chamber was set as the nondrug‐paired chamber, while the right chamber was designated as the drug‐paired chamber. (a) The exploration time of mice in Groups Ma and Ea in the left box in the baseline test and post‐test. (b) The exploration time of mice in Groups Ma and Ea in the right box in the baseline test and post‐test. *n* = 6/group. Data are shown as mean ± standard error of the mean (SEM), *p < 0.05, **p < 0.01, ***p < 0.001.

Two‐month‐old adult mice were randomly assigned into three distinct groups: the control group (Group C), receiving intraperitoneal injections of saline; the METH treatment group (Group Ma), exposed to intraperitoneal METH administration; and the exercise group (Group Ea), which underwent a 2‐week regimen of treadmill exercise intervention following intraperitoneal METH exposure. The conditioned place preference (CPP) test was used to assess METH preference, indicating the onset of dependence in both Groups Ma and Ea. Groups Ma and Ea were tested before and after METH treatment, but the CPP test was not performed after the exercise intervention. Following METH administration (post‐test phase), both Groups Ma and Ea displayed a substantial reduction in the time allocated to the left chamber (Figure 1a, *p* = 0.0003, *p* = 0.0406) and a notable increase in the time spent in the right chamber (Figure 1b, *p* = 0.001, *p* = 0.0005). These findings signify the manifestation of addictive behaviour toward METH in mice. At baseline, no differences were found in the time spent in the left (*p* = 0.4646) or right chamber (*p* = 0.9996) between the groups, indicating no inherent chamber preference. In the post‐test phase, neither group displayed similar variations in the time spent in the left chamber (*p* = 0.6691) or the right chamber (*p* = 0.5943), implying that the METH treatment regimen was impartial and that the extent of METH addiction remained comparable between the two mouse groups.

### 2.2 METH Treatment Leads to Impaired Learning and Memory Abilities in Mice

**FIGURE 2:**
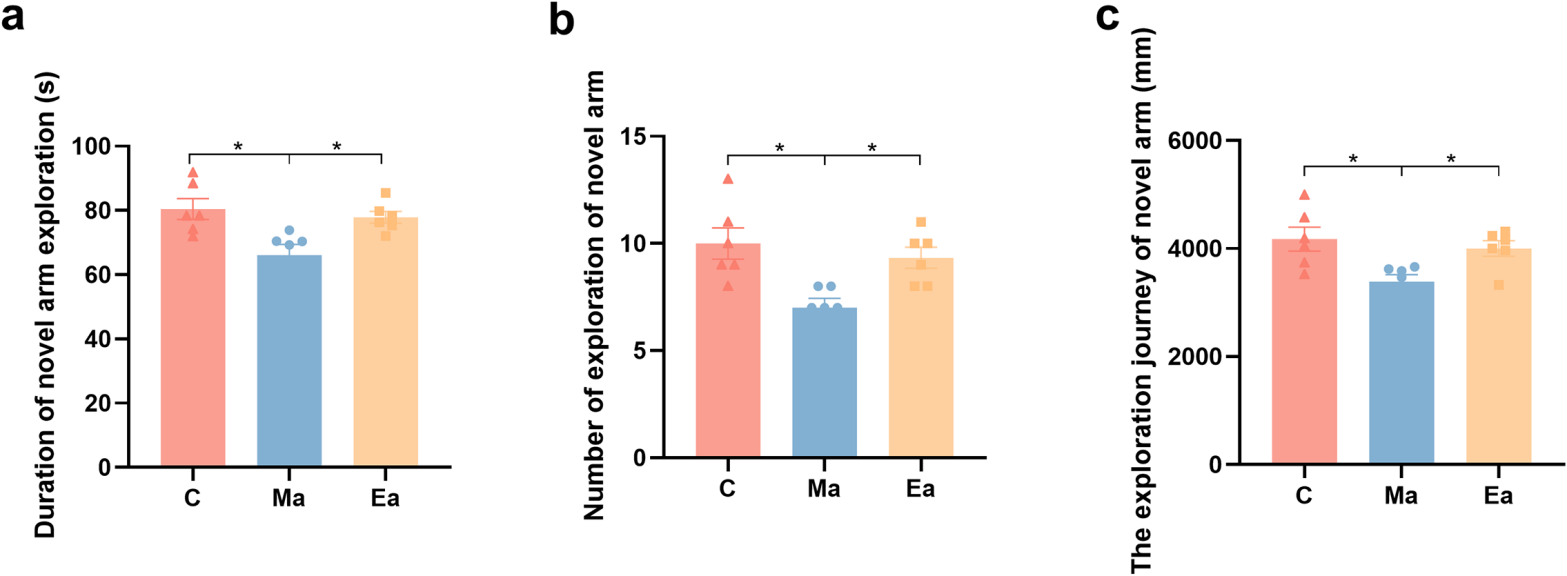
METH treatment leads to impaired learning and memory abilities in mice. (a) The duration of exploration of mice in the novel arm. (b) The number of explorations of mice in the novel arm. (c) The exploration journey of mice in the novel arm. *n* = 6/group. Data are shown as the mean ± standard error of the mean (SEM), *p < 0.05.

The Y‐maze assay is used to evaluate cognitive capabilities, learning and memory faculties and cognitive deficits in mice. Following METH treatment, a notable reduction was observed in exploration time, number of entries and distance travelled by mice in the new, unfamiliar arm when compared to Group C (*p* = 0.0321, *p* = 0.0225, *p* = 0.0419) (Figure 2). Conversely, these parameters were significantly enhanced after the exercise intervention (*p* = 0.0413, *p* = 0.0164, *p* = 0.0292) (Figure 2). These findings suggest that METH treatment impairs learning and memory in mice, while exercise can improve these deficits.

**FIGURE 3:**
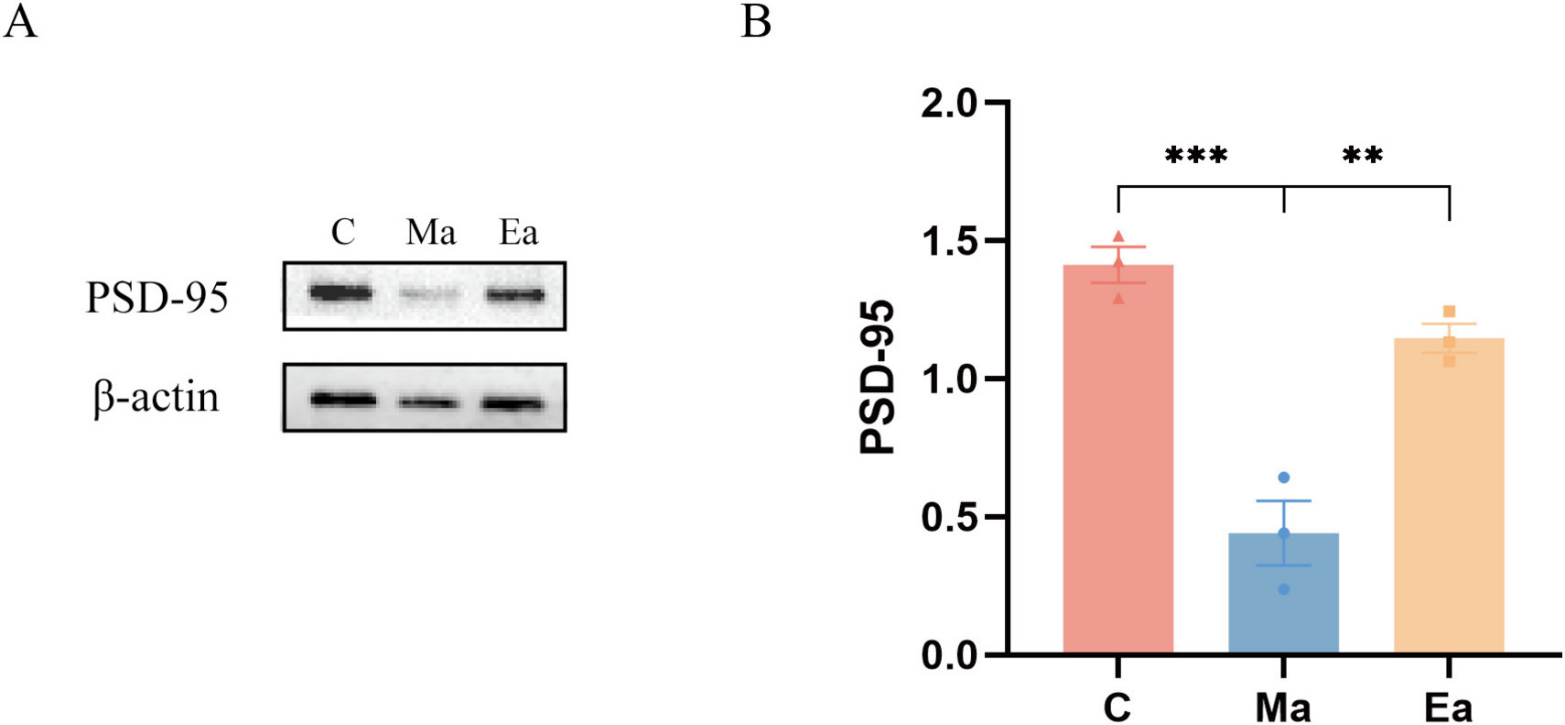
Detection results of PSD‐95 protein content in the whole brain tissue of mice in each group. *N* = 3/group. Data are shown as the mean ± standard error of the mean (SEM), *p < 0.05, **p < 0.01, ***p < 0.001.

Postsynaptic density protein‐95 (PSD‐95) plays an important role in learning and memory, especially in synaptic plasticity processes such as long‐term potentiation. The expression of PSD‐95 protein in whole brain tissues of mice in each group was detected by Western Blot. After METH treatment, the PSD‐95 protein content in the mice brain was significantly decreased (*p* = 0.0004) and significantly increased after exercise intervention (*p* = 0.0023) (Figure 3).

### 2.3 DEG Analysis

RNA extracted from the brains of mice was subjected to high‐throughput sequencing using the Illumina HiSeq X Ten platform. Nine libraries, comprising three mice from each of Groups C, Ma and Ea, were subjected to sequencing, yielding 45 to 51 million raw sequence reads per sample (Table 1). More than 97% of the reads were successfully mapped to the mouse genome.

**TABLE 1:** Summary of short‐read sequences obtained in the study.

Group	Number	Total reads	Total mapped reads
C	C1	50 203 688	49 020 701 (97.64%)
	C2	47 561 404	46 426 417 (97.61%)
	C3	49 491 992	48 294 555 (97.58%)
Ea	Ea1	50 177 594	48 894 780 (97.44%)
	Ea2	50 001 166	48 799 323 (97.60%)
	Ea3	46 820 778	45 674 198 (97.55%)
Ma	Ma1	48 748 672	47 604 935 (97.65%)
	Ma2	49 145 422	48 032 442 (97.74%)
	Ma3	50 369 334	49 153 775 (97.59%)

**FIGURE 4:**
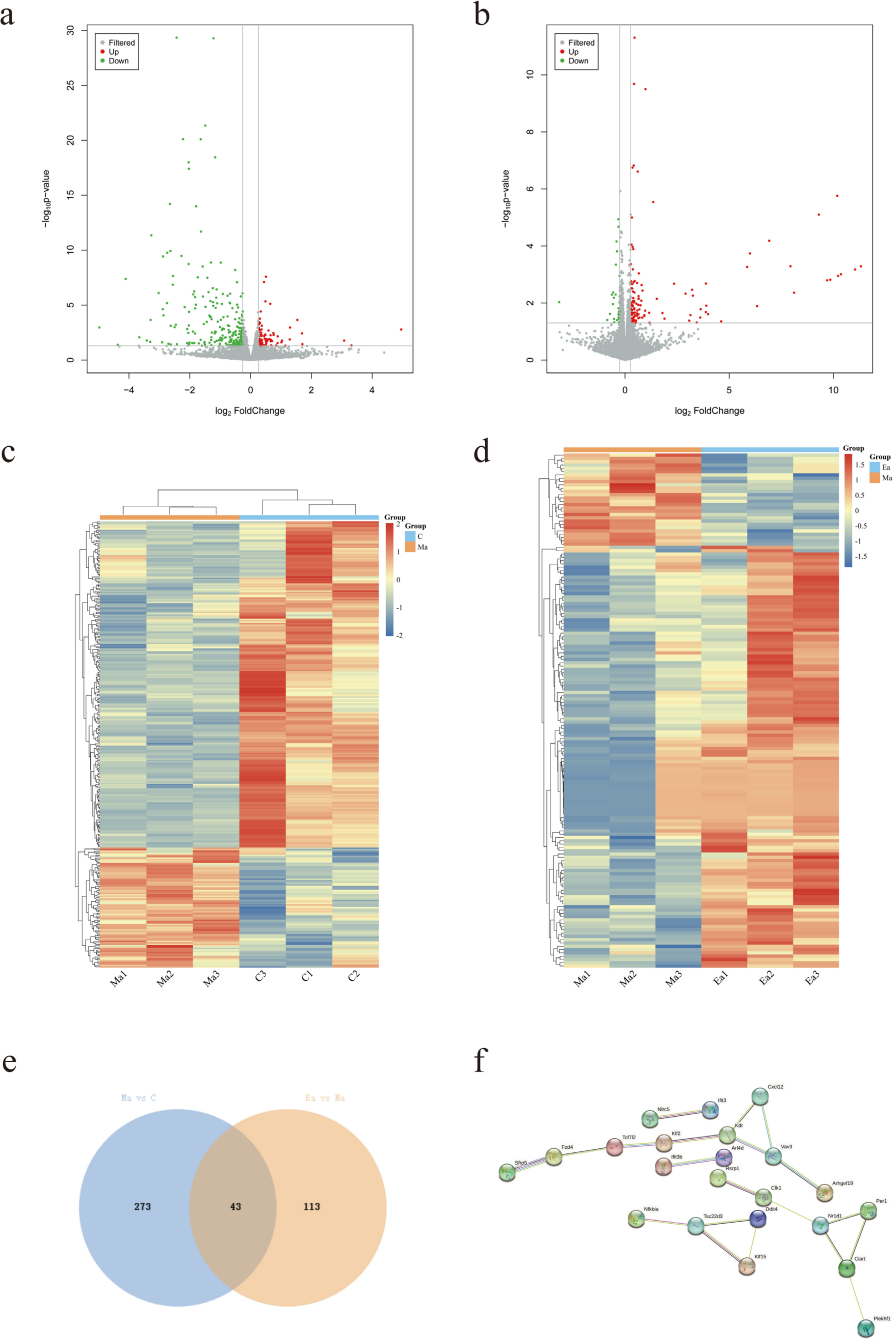
Analysis of DEGs in the brains of the three groups of mice. (a and b) The volcano plot displays the DEGs comparing Group Ma to Group C (a), as well as the DEGs comparing Group Ea to Group Ma after exercise intervention (b). DEGs were identified using threshold parameters of fold‐change > 1.2 or < 0.83 and *p*‐value < 0.05. Downregulated DEGs are represented by green dots, while upregulated DEGs are represented by red dots. (c and d) Heatmap of mRNA expression comparing Group Ma to Group C (c) and Group Ea to Group Ma (d). Red indicates relatively high expression of protein‐coding genes, while blue indicates relatively low expression of protein‐coding genes. (e) Venn diagram of DEGs after intersection analysis of DEGs in Group Ma compared with Group C and DEGs in Group Ea compared with Group Ma. (f) Construction of a PPI network for the DEGs identified in the intersection analysis using STRING.

To investigate the impact of exercise on gene expression alterations within the whole brains of METH‐treated mice, we identified differentially expressed genes (DEGs) across the three experimental groups. Compared to Group C, Group Ma exhibited 316 DEGs, encompassing 85 upregulated genes and 231 downregulated genes (Figure 4a). DEGs identified in comparison group Ma‐vs‐C are listed in Table S1. When comparing Group Ma with Group Ea, 156 DEGs were identified in Group Ea, comprising 128 upregulated and 28 downregulated genes (Figure 4b). DEGs identified in comparison group Ea‐vs‐Ma are listed in Table S2. Using unsupervised hierarchical clustering analysis, we generated a heatmap to visually represent the DEGs (Figure 4c,d).

A Venn diagram was created to show the intersection of DEGs induced by METH. This analysis revealed the presence of 43 DEGs in mice subjected to exercise (Figure 4e). DEGs identified after cross‐analysis between DEGs in Group Ma‐vs‐C and DEGs in Group Ea‐vs‐Ma are listed in Table S3. Based on these findings, we constructed a protein–protein interaction (PPI) network using STING (Figure 4f). The PPI network consisted of 41 nodes and 20 edges with an average node degree of 0.976. Furthermore, the average local clustering coefficient within the PPI network was 0.358, and the PPI enrichment *p*‐value was 2.24E−05.

### 2.4 GO and KEGG Pathway Enrichment Analyses

To gain deeper insights into the biological attributes and potential mechanistic pathways involved in the exercise‐mediated regulation of METH‐induced learning and memory impairment, we conducted Gene Ontology (GO) and Kyoto Encyclopedia of Genes and Genomes (KEGG) pathway enrichment analyses on the DEGs.

**FIGURE 5:**
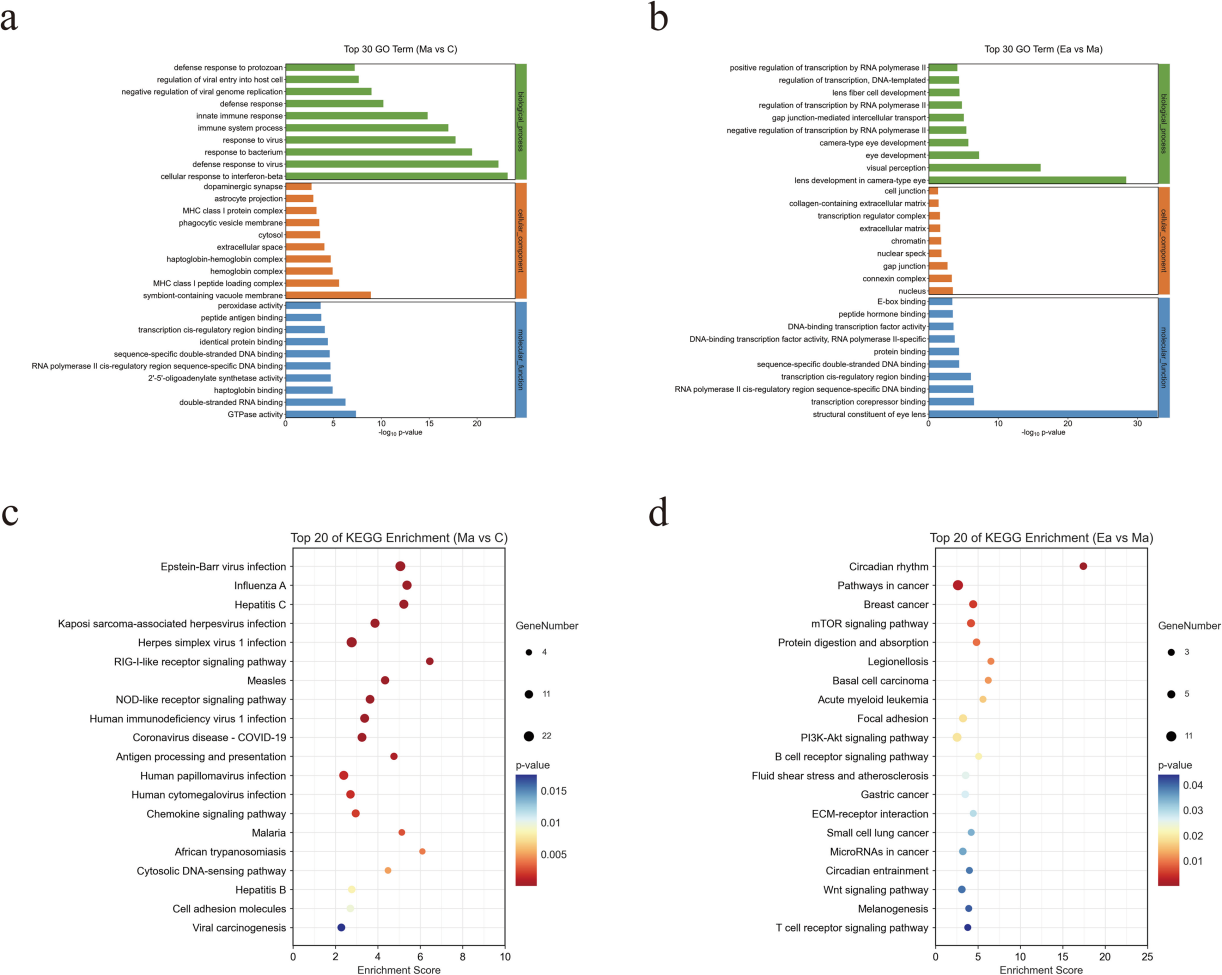
GO enrichment analysis and KEGG enrichment analysis of DEGs in Group Ma compared with Group C and DEGs in Group Ea compared with Group Ma. (a) GO enrichment analysis bar chart of the top 30 significantly enriched DEGs in Group Ma compared with Group C, including three categories: CC, BP and MF. Each category displays the top 10 significantly enriched terms. (b) GO enrichment analysis bar chart of the top 30 significantly enriched DEGs in Group Ea compared with Group Ma. (c) Bubble plot of the top 20 KEGG enrichment analyses of DEGs in Group Ma compared with Group C. (d) Bubble plot of the top 20 KEGG enrichment analyses of DEGs in Group Ea compared with Group Ma.

GO functional enrichment analysis encompasses three distinct functional categories: biological processes (BP), molecular functions (MF), and cellular components (CC). We screened GO terms exhibiting a differential gene count surpassing two in each of the BP, CC, and MF categories. Subsequently, we ranked these terms in descending order based on their respective −log10*p*‐values. The top 10 significantly enriched terms in each of the three categories are shown in Figure 5. Detailed results of GO enrichment analysis of DEGs are provided in Table S4 (Ma‐vs‐C) and Table S5 (Ea‐vs‐Ma).

Furthermore, pathway analysis was conducted on the DEGs using the KEGG database. The significance of the differential gene enrichment within each pathway entry was assessed using a hypergeometric distribution test. Detailed results of KEGG pathway enrichment analysis of DEGs are provided in Table S6 (Ma‐vs‐C) and Table S7 (Ea‐vs‐Ma). We specifically identified pathway entries associated with a differential gene count exceeding 2 and organized them based on their −log10*p*‐values. According to KEGG pathway annotation analysis, the DEGs identified in METH‐treated mice compared to controls exhibited significant involvement in pathways such as the chemokine signalling pathway, antigen processing and presentation, NOD‐like receptor signalling pathway and RIG‐I‐like receptor signalling pathway. These findings underscore the pivotal role of inflammation and the immune response following METH stimulation.

Conversely, the DEGs identified in mice subjected to exercise intervention in comparison to METH‐treated mice were significantly associated with pathways including the PI3K‐Akt, mTOR and Wnt signalling pathways. These outcomes suggest that the beneficial effects of exercise may be mediated through the regulation of signal transduction, growth and metabolism.

**FIGURE 6:**
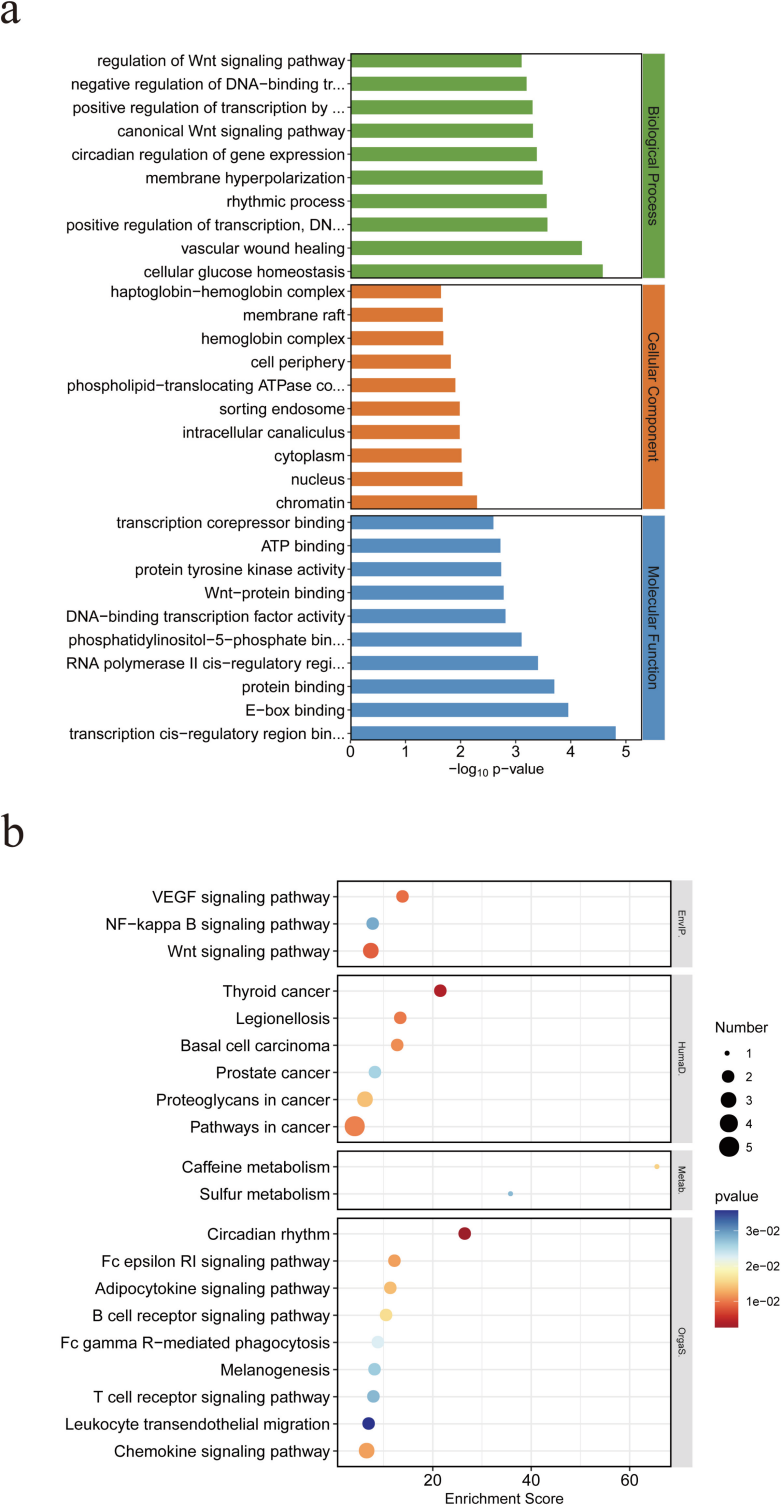
Pathway enrichment analysis of DEGs after cross‐analysis between DEGs in Groups Ma and C and DEGs in Groups Ea and Ma. (a) Bar chart of the top 30 significantly enriched GO enrichment analyses, including three categories: CC, BP and MF. (b) Top 20 KEGG enrichment analysis bubble chart.

To elucidate the biological characteristics of exercise in the context of METH‐induced memory impairment, we conducted pathway enrichment analysis by intersecting the DEGs from Group Ea with those from Group Ma and the DEGs from Group Ma with those from Group C (Figure 6).

Figure 6 shows the top 10 significantly enriched terms within the BP, CC and MF categories. Moreover, in‐depth KEGG pathway analysis revealed that exercise is likely to exert its ameliorative effects through pathways such as the chemokine signalling pathway, Wnt signalling pathway and NF‐kappa B signalling pathway. Detailed information on these pathways is provided in Table 2.

**TABLE 2:** The top 20 pathways of KEGG enrichment analysis of DEGs after cross‐analysis.

		Pathway	Count	*p*	Gene
1	mmu04710	Circadian rhythm	2	0.002582	Nr1d1; Per1
2	mmu05216	Thyroid cancer	2	0.00391	Ret; Tcf7l2
3	mmu04310	Wnt signalling pathway	3	0.008489	Fzd4; Sfrp5; Tcf7l2
4	mmu04370	VEGF signalling pathway	2	0.009396	Kdr; Pla2g4e
5	mmu05134	Legionellosis	2	0.010031	Clk1; Nfkbia
6	mmu05200	Pathways in cancer	5	0.010317	Cxcl12; Fzd4; Nfkbia; Ret; Tcf7l2
7	mmu05217	Basal cell carcinoma	2	0.011017	Fzd4; Tcf7l2
8	mmu04062	Chemokine signalling pathway	3	0.012037	Cxcl12; Nfkbia; Vav3
9	mmu04664	Fc epsilon RI signalling pathway	2	0.012044	Pla2g4e; Vav3
10	mmu04920	Adipocytokine signalling pathway	2	0.013847	Adipor2; Nfkbia
11	mmu05205	Proteoglycans in cancer	3	0.013996	Fzd4; Kdr; Vav3
12	mmu00232	Caffeine metabolism	1	0.015194	Xdh
13	mmu04662	B cell receptor signalling pathway	2	0.016155	Nfkbia; Vav3
14	mmu04666	Fc gamma R‐mediated phagocytosis	2	0.022587	Pla2g4e; Vav3
15	mmu05215	Prostate cancer	2	0.025896	Nfkbia; Tcf7l2
16	mmu04916	Melanogenesis	2	0.026384	Fzd4; Tcf7l2
17	mmu00920	Sulfur metabolism	1	0.027687	Papss2
18	mmu04660	T cell receptor signalling pathway	2	0.027871	Nfkbia; Vav3
19	mmu04064	NF‐kappa B signalling pathway	2	0.028881	Cxcl12; Nfkbia
20	mmu04670	Leukocyte transendothelial migration	2	0.035796	Cxcl12; Vav3

### 2.5 Validation of RNA‐Seq Results by RT–qPCR

**FIGURE 7:**
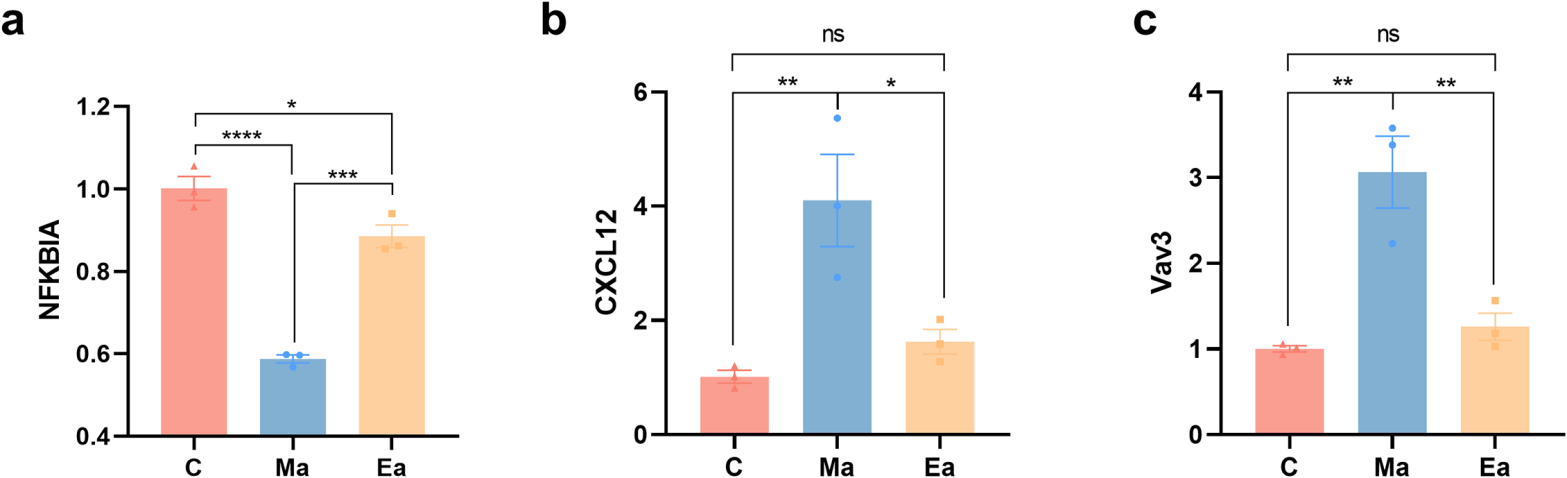
Bar graph of the results of measuring the expression of NFKBIA, Cxcl12 and Vav3 in the brains of the three groups of mice by real‐time quantitative fluorescence PCR. *N* = 3/group. Data are shown as the mean ± standard error of the mean (SEM), *p < 0.05, **p < 0.01, ***p < 0.001, ****p < 0.0001.

To validate the expression profile of the DEGs identified through RNA‐seq, we specifically focused on the DEGs within the chemokine signalling pathway, namely, NFKBIA, Cxcl12 and Vav3, which emerged from the enriched KEGG pathway after cross‐analysis. These DEGs were carefully selected for qPCR analysis (Figure 7).

The results of real‐time quantitative PCR (RT–qPCR) aligned harmoniously with the RNA‐Seq data, underscoring the reliability and a certain degree of accuracy inherent in the RNA‐Seq finding.

## 3 Discussion

METH, an amphetamine‐type central stimulant, poses a significant risk to the CNS by inducing neurotoxic effects [10]. Reports have highlighted the detrimental impact of METH use on the CNS, encompassing symptoms such as agitation, irritability, anxiety, depression, cognitive deterioration and mental disorders. In extreme cases, high‐dose ingestion can even lead to fatal outcomes [11, 12]. Currently, treatment options for METH addiction and CNS neurotoxic effects are limited. Recent research has underscored the potential of physical exercise as a viable intervention for drug addiction, especially METH addiction [13]. Studies have shown that exercise can ameliorate the symptoms of depression and anxiety, mitigate addictive behaviours, enhance sleep quality, bolster learning and memory capacities and alleviate cognitive impairments frequently observed in METH users [14]. Previous investigations have also demonstrated that exercise can mitigate metabolic disturbances precipitated by METH dependence [15]. Research on the dose–response relationship between exercise and METH craving suggests that moderate‐intensity acute exercise can reduce cravings and improve inhibitory control in METH‐dependent individuals [16]. Exercise may improve drug addiction by modulating CNS neurochemicals, altering signal transduction in reward pathways, and reversing METH‐induced chromatin changes [6, 17].

Previous studies have highlighted the positive effects of physical exercise, which encompasses the regulation of neurochemical equilibrium, attenuation of oxidative stress, fortification of the blood–brain barrier, promotion of neurogenesis and augmented production of glial cells to mitigate neurological damage stemming from METH exposure, thereby ameliorating addiction [7]. Understanding how exercise mitigates METH‐induced learning and memory impairments can improve its use as a therapeutic approach for METH addiction.

This study aimed to assess the impact of treadmill exercise intervention on METH‐induced deficits in learning and memory function in mice while endeavouring to identify potential mechanistic pathways. These findings revealed that exercise effectively enhanced the learning and memory functions of METH‐treated mice. Specific targets, such as NFKBIA, Cxcl12 (C‐X‐C motif chemokine ligand 12) and Vav3, along with key signalling pathways like chemokine, Wnt and NF‐kappa B, may mediate the effects of exercise on METH‐induced learning and memory impairments. These pathways are intrinsically linked to neuroregulation and endocrine modulation, among other processes.

The CPP test measured METH dependence in mice by assessing changes in their preference for drug‐related environments. Compared with self‐administration experiments, which are technically demanding and time‐consuming, the CPP test is easy to operate and highly sensitive. The results of the CPP test unequivocally indicated that after 7 days of drug injection, mice developed dependence on METH. Repeated exposure to METH induces neurodegenerative changes, including cognitive impairments, memory dysfunction, learning difficulties and symptoms of anxiety and depression [4, 18].

The Y‐maze task, employed to evaluate learning and memory abilities as well as cognitive deficits in mice, enables them to explore the three arms of the maze, involving various brain regions such as the hippocampus, basal forebrain and prefrontal cortex. Compared with other cognitive tests, such as the Morris water maze test, the Y‐maze is easier to operate and avoids the possible interference of long‐term training and stress response to the water environment on the experimental results. The Y‐maze results showed that METH‐exposed mice spent less time exploring the novel arm. Conversely, physical exercise interventions significantly extended the exploration time. PSD‐95 is involved in synaptic plasticity, synaptic transmission and signal transmission between neurons. After METH treatment, the PSD‐95 protein content in the whole brain tissue of mice decreased significantly and increased significantly after exercise intervention. This compelling evidence underscores the capacity of treadmill exercise to enhance the learning and memory abilities of mice while mitigating the cognitive deficits induced by METH.

To elucidate the molecular mechanisms underlying the influence of exercise on METH‐induced learning and memory impairments, transcriptional analysis was conducted in the brain tissues of each group of mice. Particular emphasis was placed on the effect of exercise intervention on changes in neurochemical substances induced by METH treatment. KEGG pathway enrichment analysis revealed that the DEGs between METH‐treated mice and the control group were significantly linked to pathways such as chemokine signalling, antigen processing and presentation, NOD‐like receptor signalling and RIG‐I‐like receptor signalling. These findings underscore the pivotal role of inflammation and the immune response following METH stimulation. METH increases the expression of glial fibrillary acidic protein in the striatum and cortex. METH induces microglial activation in multiple brain regions, including the hippocampus, striatum and cortex, leading to an increased production and secretion of pro‐inflammatory cytokines, which in turn contribute to neurodegenerative changes [19]. METH exposure causes significant microglial changes in limbic structures, with hypertrophy in the hippocampus and amygdala [20]. In METH‐exposed rats, severe neurodegeneration occurs in the thalamus, along with extensive microglial activation [21]. Conversely, the DEGs identified in mice subjected to exercise intervention in comparison to METH‐treated mice were notably associated with pathways including the PI3K‐Akt, mTOR and Wnt signalling pathways. These results suggest that the beneficial effects of exercise can be attributed to the regulation of signal transduction, growth, metabolism and other related processes.

In this study, a significant downregulation of Nfkbia expression was observed in the brains of mice following METH treatment, whereas exercise intervention resulted in its upregulation. Nfkbia serves as a pivotal inhibitor of NF‐κB (the nuclear factor kappa B) signalling pathway [22]. NF‐κB is a transcription factor of paramount importance that governs processes such as cell survival, development, immune responses, apoptosis and inflammation. Perturbations in NF‐κB activation have been linked to memory impairment and enhancement [23]. Notably, NF‐κB within the CNS is instrumental in regulating synaptic plasticity as well as learning and memory [23, 24]. Exercise intervention may improve METH‐induced memory and learning impairment by upregulating the expression of NFKBIA and inhibiting abnormal NF‐κB signalling activity.

CXCL12 plays a vital role in various aspects of brain development within hippocampal neurons, including cell migration and axon pathfinding [25, 26, 27]. The primary receptor for CXCL12 in the brain is CXCR4(C‐X‐C Motif Chemokine Receptor 4). Studies have highlighted that blocking CXCR4 in the cortical neurons of rats can lead to reduced survival and induce cell death through intrinsic apoptotic mechanisms [28]. Notably, repeated exposure to cocaine has been associated with increased CXCL12 gene expression in the ventral tegmental area [29]. This suggests that CXCL12 may be involved not only in the development of the brain but also in neuroplastic changes associated with addictive behaviours and thus may serve as a potential biomarker or therapeutic target for addiction‐related cognitive dysfunction.

Vav3 plays a pivotal role in various critical processes during central nervous system development. These processes include regulation of cell division [30], guidance of migrating neurons [31], involvement in oligodendrocyte maturation, myelination, remyelination and oligodendrocyte differentiation [32] and participation in neuronal differentiation [33]. Notably, primary hippocampal neurons derived from Vav3‐deficient embryonic animals exhibit significantly increased axonal length and complexity along with an elevated number of structural synapses in in vitro settings [33]. Our study showed that Vav3 expression changed significantly after exercise intervention, suggesting that it may play an important role in the regulation of synaptic plasticity and improvement of learning and memory.

In this study, we found that treadmill exercise could alleviate the cognitive impairment induced by METH treatment to a certain extent and revealed the potential molecular mechanism through transcriptome analysis. However, there are certain limitations to this study, one of which is that the transcriptome analysis is based on the whole brain tissue rather than specific brain regions. While whole‐brain analysis can provide a holistic gene expression pattern, it may mask specific expression changes between different brain regions. For example, regions such as the cerebral cortex, hippocampus and striatum have different functions in learning, memory and addictive behaviour, and they may exhibit different transcriptome characteristics in response to METH or exercise intervention. Future studies can further explore the unique roles of different brain regions in exercise intervention by performing transcriptome analysis on specific brain regions, as well as in combination with other techniques (such as single‐cell RNA sequencing and spatial transcriptome analysis) to better understand the mechanism of the effect of exercise on METH‐induced cognitive impairment.

In the present study, DEGs in the brains of mice were identified using RNA‐seq, followed by a comprehensive pathway enrichment analysis. Subsequently, the mRNA expression levels of these DEGs, including NFKBIA, CXCL12 and Vav3, in the brains of mice within each group were meticulously assessed using RT–qPCR experiments. While the results suggest that treadmill exercise intervention has an ameliorative effect on METH addiction in mice, likely mediated through these specific targets, it is paramount to acknowledge that further research is warranted to gain a comprehensive understanding of the intricate mechanisms at play.

## 4 Conclusion

In this study, we performed a mouse brain transcriptome sequencing analysis with experimental validation to investigate the advantageous effects of exercise on METH‐induced memory impairment and discern its potential therapeutic mechanisms. The behavioural tests conducted in this investigation unequivocally affirmed that physical exercise can ameliorate METH addiction behaviour, memory impairment and cognitive deficits.

The findings obtained from transcriptome sequencing analysis indicated that exercise may exert beneficial effects on memory impairments and cognitive deficits induced by METH by modulating various pathways, including gastric acid secretion, circadian entrainment and the oxytocin signalling pathway. Furthermore, specific targets, such as NFKBIA, CXCL12 and Vav3, are potential mediators of these therapeutic effects. These findings collectively hint at the prospect of exercise interventions and clinical research trials involving individuals with METH addiction, which may serve as instrumental endeavours to delineate and endorse the potential therapeutic efficacy of exercise in this context.

## 5 Materials and Methods

### 5.1 Reagents

The Sichuan Police College–Sichuan Provincial Key Laboratory of Intelligent Policing generously supplied METH (> 99% purity).

### 5.2 Animals

Eighteen male C57BL mice, aged 8 weeks, were procured from Chengdu Dossy Experimental Animal Co. Ltd. (Chengdu, China). Mice were housed in a specific pathogen‐free animal facility at the Sichuan Key Laboratory of Sports Medicine, Chengdu Sport University. They were subjected to controlled environmental conditions, including a 12‐h light–dark cycle, a temperature maintained at (21 ± 2)°C and ad libitum access to food and water. Following 1 week of acclimatization and adaptive feeding, the mice were randomly assigned into three distinct groups, each comprising six mice. They were housed in separate cages, with three mice per cage. The three groups were as follows: Group C (*n* = 6), Group Ma (*n* = 6) and Group Ea (*n* = 6).

All animal experiments adhered strictly to the guidelines stipulated in the US National Research Council's “Guide for the Care and Use of Laboratory Animals” (8th Edition, 2011). Additionally, the experimental protocol received the requisite approval from the Ethics Committee of Chengdu Sports Institute (Approval No. {2022} 56).

### 5.3 Experimental Protocol

**FIGURE 8:**
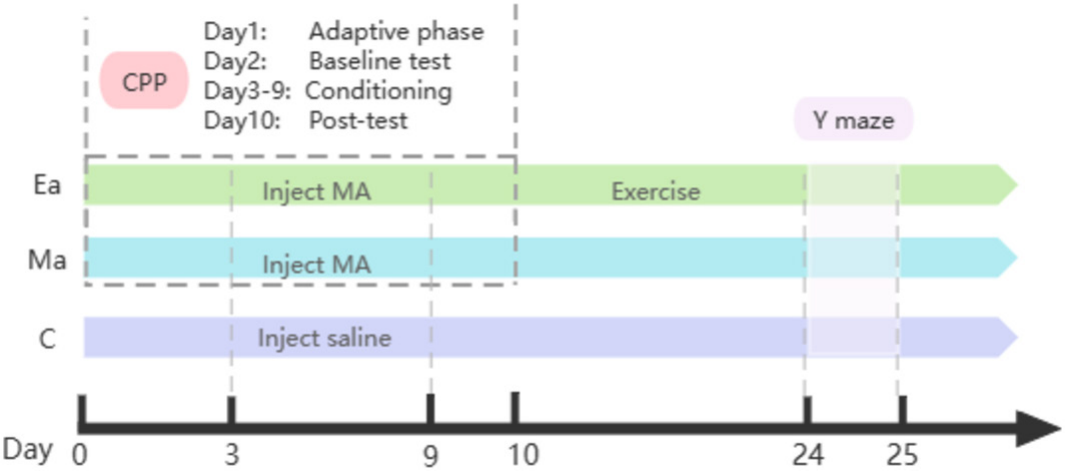
Experimental procedures for exercise intervention in METH‐addicted mice.

The experimental protocol and the intervention plan are shown in Figure 8. Consistent with prior investigations [34, 35], METH was administered to both Groups Ma and Ea via intraperitoneal injection at a dose of 1 mg/kg. Prior to administration, METH was dissolved in physiological saline to a concentration of 1 mg/mL. Concurrently, Group C received intraperitoneal injections of an equivalent volume of normal saline. These drug injections were administered at 24‐h intervals over a continuous seven‐day period, with simultaneous conduction of the CPP verification experiment.

After the CPP test, aerobic exercise was initiated for Group Ea mice on a treadmill. In alignment with previously established protocols [36, 37], a treadmill exercise regimen (SA101, Jiangsu Science Biological Technology Co. Ltd., Jiangsu, China) incorporated a slope of 0° and included speeds of 8, 10 and 12 m/min on the 1st to 3rd day of the exercise intervention, and the speed was gradually increased to adapt to the environment. From the 4th to the 14th day, the mice engaged in aerobic exercise at a consistent speed of 12 m/min, with each session lasting 60 min. This regimen persisted for a total of 2 weeks. Avoid excessive fatigue or stress reactions that may be caused by high‐intensity exercise. The exercise sessions for Group Ea mice were scheduled in the afternoon, commencing at 2 PM. In instances where the mice exhibited inactivity, no electrical stimulation was applied; instead, gentle tail tapping was employed to encourage them to resume the exercise. Groups Ma and C were maintained under routine cage conditions without any intervention.

Following completion of the exercise intervention, all three groups of mice underwent the Y‐maze test. Subsequently, the mice from each group were anaesthetised and humanely euthanized by the administration of 0.5% pentobarbital sodium (50 mg/kg). Their brains were then carefully extracted, placed in cryovials, promptly submerged in liquid nitrogen and subsequently preserved at −80°C to facilitate molecular biology analysis.

### 5.4 Conditioned Place Preference

The CPP test was used to assess psychotropic drug dependence in mice. The CPP apparatus (SA213, Jiangsu Science Biological Technology Co. Ltd., Jiangsu, China) comprised a shuttle box measuring 210 × 170 × 205 mm equipped with an infrared receiving system connected to a computer‐based data acquisition system. The shuttle box was divided into two chambers: a white compartment (right chamber) and a black compartment (left chamber). In the experimental configuration, the right chamber, characterized by white walls, served as the drug‐paired chamber, whereas the left chamber, featuring black‐striped walls, functioned as a nondrug‐paired chamber. A partition separating the two compartments can be increased or lowered. Each compartment was equipped with lighting and an overhead infrared‐receiving system. The infrared receiving system was linked to a computer via an electrical signal converter, enabling recording and subsequent analysis of the time spent by the mice in each chamber.

The CPP experiment encompassed four distinct phases (Figure 7). On the initial day (adaptive phase), the partition was removed, allowing the unrestricted exploration of both compartments for a duration of 10 min. Subsequently, on the following day (baseline test stage), a baseline assessment was conducted, wherein the mice were provided 10 min to freely explore both compartments. The time spent by the mice in each compartment, as well as the distance travelled, was meticulously recorded. During the conditioning phase, from the 3rd to the 9th day, the mice received daily METH injections, with placement in the drug‐paired compartment, and saline injections, with placement in the nondrug‐paired compartment, administered at an 8‐h interval between injections. After each injection, the mice were immediately confined to one of the two compartments for 30 min. Finally, on the 10th day (post‐test phase), no drug injections were administered, permitting the mice to once again explore both chambers freely for 10 min, with the duration of their stay recorded. An increase in the time spent by the mice in the drug‐paired compartment during the post‐test phase compared to the baseline test phase signified successful experimental modelling, indicating the development of METH dependence in the mice.

### 5.5 Y‐Maze

The Y‐maze serves as an assessment tool to evaluate the memory and cognitive abilities of mice [38, 39]. The Y‐maze configuration consisted of three identical arms, each subtending at an angle of 120°. The dimensions of each arm were 350 × 45 × 200 mm (length × width × height), and a movable partition was positioned at the central junction of the maze. Within the Y‐maze, the three arms are designated as the novel arm, start arm and other arm. During each trial, the mice were introduced into the Y‐maze from the start arm. Visual markers in the form of triangles attached to the inside of the novel arm and circles attached to the other arm aid in distinguishing them. A camera lens placed 1.5 m above the maze captured the entire process. The Y‐maze experiment encompassed two stages: training and testing.

During the training period (first stage), the novel arm was blocked using a partition. The mice were introduced into the Y‐maze via the start arm and allowed to explore both the start arm and the other arms freely for a duration of 10 min. Following this training session, mice were returned to their respective housing cages.

The second stage, the testing period, occurs 1 h after the training phase. During this stage, the partition obstructing the novel arm is removed. The mice are placed into the Y‐maze via the start arm and allowed to explore the Y‐maze freely for 5 min. During this phase, the time spent by the mice in each arm and the number of transitions between the arms are recorded as measures of cognitive performance.

### 5.6 RNA Isolation and Library Preparation

Total RNA was extracted from the whole brain tissues of each group of mice using TRIzol reagent following the manufacturer's instructions. The obtained RNA samples were used to construct sequencing libraries. RNA quality and quantity were determined using a NanoDrop 2000 spectrophotometer (Thermo Scientific, USA). Additionally, the integrity was assessed using an Agilent 2100 Bioanalyzer (Agilent Technologies, Santa Clara, CA, USA). Libraries were constructed using the TruSeq Stranded mRNA LT Sample Preparation Kit (Illumina). Transcriptome sequencing was conducted by OE Biotech Co. Ltd. (Shanghai, China).

### 5.7 RNA Sequencing and Differentially Expressed Genes Analysis

The libraries were sequenced on the Illumina HiSeq X Ten platform, producing 150 bp paired‐end reads. To ensure data quality, the raw reads in FASTQ format underwent an initial processing step using Trimmomatic [40], which involved the removal of low‐quality reads to yield clean reads.

For further analysis, clean reads were subjected to several steps. The FPKM (the reads per kilobase of exon per million fragments mapped) [41] of each gene was computed using Cufflinks [42], and the read count for each gene was determined using HTSeqcount [43]. Differential gene expression analysis was performed using the DESeq R package (2012). A *p* < 0.05, fold‐change > 1.2 or < 0.83 was set as the threshold for significant differential expression.

Hierarchical cluster analysis of DEGs was performed to illustrate the expression patterns across different groups and samples. Additionally, GO enrichment and KEGG pathway enrichment analyses of DEGs were conducted using R based on hypergeometric distribution [44] To investigate the related target proteins influenced by exercise and their impact on METH‐induced learning and memory deficits, a PPI network was constructed. Network analysis was performed using the online STRING 11.5 platform (https://string‐db.org/).

### 5.8 Western Blot Analysis

Total protein was extracted from the whole brain tissue of each group of mice using RIPA buffer supplemented with protease inhibitors. Protein concentration was quantified using the BCA assay (Thermo Fisher Scientific). Equal amounts of protein (20 μg) were separated by 12.5% SDS‐PAGE and transferred to a PVDF membrane (Millipore, IPVH00010). The membrane was blocked with 5% non‐fat milk at room temperature for 2 h, followed by overnight incubation at 4°C with primary antibodies. After washing the membrane with TBST, secondary antibodies were incubated at room temperature for 2 h. Protein bands were visualized using ECL chemiluminescent substrate (Abbkine, BMU102‐CN) and detected with a chemiluminescence imaging system (BIO SPECTRUM). Band intensities were analysed using ImageJ software to quantify the relative expression levels of proteins. The antibodies and their dilutions were as follows: PSD‐95 (1:1000, Cell Signaling Technology, #3450) and β‐actin (1:10 000, Affinity, #AF7018).

### 5.9 Quantitative Real‐Time Polymerase Chain Reaction Analysis

To validate the DEGs identified from the RNA‐Seq results, RT–qPCR was conducted using GAPDH as an internal reference gene. Gene amplification was performed in a 20‐μL reaction volume. Relative gene expression was calculated using the 2^−ΔΔCT^ method, and amplification efficiency was assessed based on amplification and melting curves.

Primers for the target genes were synthesized by Sangon Biotech (Shanghai, China), and the gene and primer sequences are listed in Table 3. Initially, total RNA (1 μg) was reverse transcribed into cDNA using the Hifair III 1st Strand cDNA Synthesis Kit, following the manufacturer's protocol. Subsequently, RT–qPCR was performed using the Hifair qPCR SYBR Green Master Mix Kit and QuantStudio 6 Flex Real‐Time PCR System.

**TABLE 3:** Primer sequences of target genes.

Genes	Forward primer	Reverse primer	Accession number
GAPDH	AGGTCGGTGTGAACGGATTTG	GGGGTCGTTGATGGCAACA	NM_008084
Nfkbia	CCTAGACCCAGGCATTTTACTG	AAAAATCCCACAAACATGAACC	NM_010907
CXCL12	GCTCCCTTGGTTCAGAAAATTG	TCACCAGACAGGTGCCATCA	NM_001012477
Vav3	ATGGAGCCGTGGAAGCAGTG	TCCGCCTTCATCAAGTCTTC	NM_020505

### 5.10 Statistical Analysis

The results are presented as the mean ± SEM. To assess the normal distribution of the data, SPSS software (version 22.0) was used. Statistical analyses comparing multiple groups were conducted using one‐way analysis of variance. In cases where data did not follow a normal distribution for two or three groups, the Mann–Whitney U test and Kruskal–Wallis test were employed, respectively. Graphs were generated using GraphPad Prism 8.4.2 software (La Jolla, CA, USA). Statistical significance was set at *p* < 0.05.

### Author Contributions

**Qiuyue Huang:** writing – original draft, visualization. **Jisheng Xu:** investigation, methodology. **Xuejie Zhang:** data curation, resources. **Changling Wei:** conceptualization, supervision. **Tianzhen Zheng:** methodology, validation. **Xin Liang:** writing – review and editing. **Xue Li:** project administration.

## Ethics Statement

The experimental protocol received the requisite approval from the Ethics Committee of Chengdu Sports Institute (Approval No. {2022} 56).

## Consent

The authors have nothing to report.

## Conflicts of Interest

The authors declare no conflicts of interest.

## Supporting information


**Table S1:** DEGs identified in comparison group Ma‐vs‐C.

**Table S2:** DEGs identified in comparison group Ea‐vs‐Ma.

**Table S3:** DEGs identified after cross‐analysis between DEGs in Group Ma‐vs‐C and DEGs in Group Ea‐vs‐Ma.

**Table S4:** Detailed information of GO enrichment of DEGs in group Ma‐vs‐C.

**Table S5:** Detailed information of GO enrichment of DEGs in group Ea‐vs‐Ma.

**Table S6:** Detailed information of KEGG pathway enrichment of DEGs in group Ma‐vs‐C.

**Table S7:** Detailed information of KEGG pathway enrichment of DEGs in group Ea‐vs‐Ma.

## Data Availability

The datasets generated and/or analysed during the current study are available at NCBI project PRJNA1029934 (https://www.ncbi.nlm.nih.gov/sra/?term=PRJNA1029934) with accession number of nine objects (SRR26445921, SRR26445920, SRR26445919, SRR26445918, SRR26445917, SRR26445916, SRR26445915, SRR26445914 and SRR26445913). Any reasonable requests are available from the corresponding author.
